# Modelling stomatal mechanics: a critical review

**DOI:** 10.1111/nph.70826

**Published:** 2025-12-12

**Authors:** Nathanael Y. H. Tan, Jodie V. Armand, Julie E. Gray, Andrew J. Fleming

**Affiliations:** ^1^ Plants, Photosynthesis and Soils, School of Biosciences University of Sheffield Western Bank Sheffield S10 2TN UK

**Keywords:** biomechanics, cell geometry, finite element method, mechanical advantage, modelling, plant cell wall, stomata

## Abstract

The biomechanics of stomatal movements have fascinated scientists for almost 150 yr, yet we still lack a conclusive and coherent mechanistic understanding of the process. In this review, we present a framework that allows critical insight into the state of knowledge of stomatal biomechanics, with a focus on modelling approaches. We apply the framework in two ways. First, contextualising the history, we show how the nature and function of models of stomatal mechanics have evolved. Second, we use the framework to appraise three key features of extant models: cell wall mechanical properties, guard cell shape, and the role of surrounding epidermal cells. We evaluate the empirical origin and model representations of these features, summarising how each is currently thought to explain stomatal function, while also identifying limitations in our understanding. We propose that a better appreciation of gaps in knowledge in the empirical domain, particularly the actual shifts in cell shape during stomatal response, combined with careful reinterpretation of existing data, will lead to new insight and a more complete understanding of stomatal mechanics.

## Introduction

Stomata are microscopic pores on the aerial surfaces of plants formed between pairs of guard cells (GCs) that dynamically change in size and shape to regulate pore size, and thus the rate of photosynthetic gas exchange and plant water loss. As key innovations in plant evolution (Raven, 2002), major players in the global cycling of carbon and water (Hetherington & Woodward, 2003), and a potential target for engineering more water‐efficient crops (Lawson & Blatt, 2014; Gray & Dunn, 2024), understanding how stomata function has importance at both a fundamental and applied level. As a result of extensive research over many decades, we have an advanced understanding of the signalling pathways that initiate the process of pore opening/closure, and of the co‐ordinated action of ion channel activities that underpin the changes in turgor pressure that ultimately provide the forces that drive cell expansion/deflation (Kim *et al*., 2010; Kollist *et al*., 2014). The actual opening/closure of the pore, however, arises from the interaction between forces generated and distributed within and around the cellular stomatal complex; it is ultimately a mechanical process. Although significant advances have been made in this area over the last decade, there remain major gaps in our understanding, which we will highlight in this review.

**Fig. 1:**
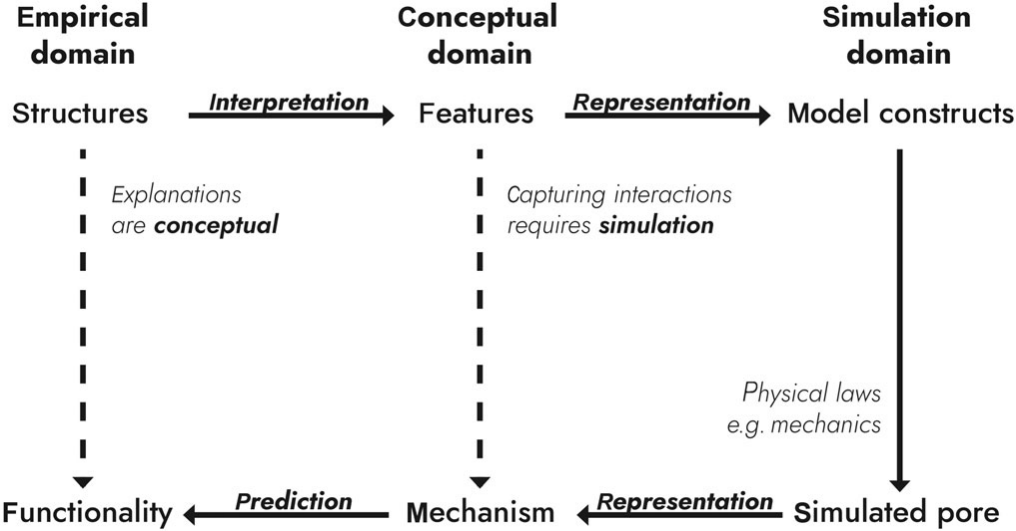
Framework for the relationship between observations, mechanisms, and models of stomatal mechanics. The simulation domain (model) depends upon the selection of conceptual features proposed to play a role in stomatal opening/closing. In the conceptual domain, features are interpreted from observations and experiments designed to capture data on selected structures proposed to be physically important in cellular mechanics (empirical domain). The outputs of simulations provide insight into the mechanism of the system and, eventually, the functionality of observed structures within the systems. Solid arrows indicate the sequential practical steps taken in the modelling process, with dashed arrows indicating the eventual advances in insight provided by the modeling approach.

To approach this problem, we first describe a general framework that outlines the relationship between empirical (observable) data, conceptual notions of mechanisms, and the simulation models used to study them (Fig. 1). We then use the framework to chart progress in our understanding of stomatal mechanics by assessing both old and new models. From critical analysis, we identify broad elements of extant models, which we believe warrant further investigation to achieve a fuller understanding of how stomata work at a mechanistic level.

Stomata exist in a wide variety of forms (Spiegelhalder & Raissig, 2021; Rudall, 2023), but in this review, we focus on the common eudicot ‘kidney‐shaped’ stomata. First, this is where most research in this area has historically been performed; second, it is the platform where, considering *Arabidopsis* in particular, the technical approaches for testing hypotheses generated by models are most developed. We bring in examples on biomechanics from evolutionary distinct forms of stomata as appropriate, but these are not the focus of this review.

## A framework for stomatal mechanics

The goal of this review was to evaluate the features and mechanisms underlying the physical movement of stomata (which is the aspect of stomatal function covered by the literature). Models are an essential component of this effort. To allow a comparison of published models, and to enable judgement of the progress made, we have generated a conceptual framework that distils the main elements of the modelling process (Fig. 1). In this framework, we view models as assemblies of features in the *conceptual domain*. These features are taken from observed structures in the *empirical domain*. Incorporation of these structures into a model requires interpretation of their function. To investigate the consequences of interactions between the features, it is necessary to ‘cast them into mathematical form’ using physical laws (Phillips *et al*., 2013), creating the simulation domain. The outputs of the simulations provide a representation of the system which, hopefully, provides insight into the mechanism. If the simulation allows prediction of system behaviour, then this provides validation of the model utility; that is, a level of insight into biological function has been achieved.

**Fig. 2:**
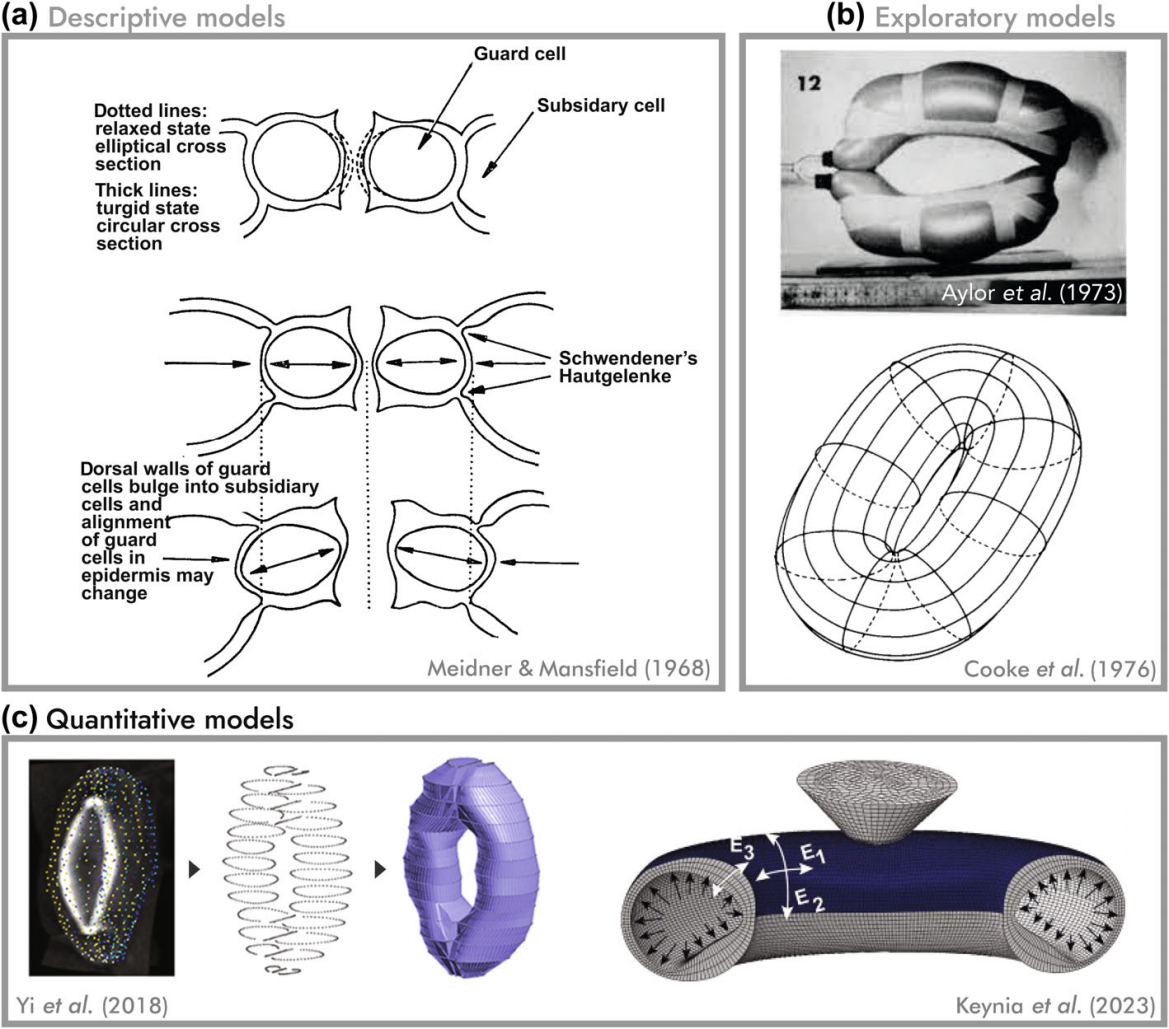
Classification of stomatal models according to their purpose. (a) Descriptive models are the simplest form of models, which illustrate the scope of the model, its features, and their relationships. However, lacking simulation, they are unable to demonstrate cause and effect. Illustrations shown are from Meidner and Mansfield (1968, every effort has been made to contact the copyright holder) but are inspired from the century prior (Schwendener, 1881). (b) Exploratory models such as Aylor *et al*. (1973) (reproduced with permission) and Cooke *et al*. (1976) (reproduced with permission from ASABE: Cooke *et al*. (1976). A Finite Element Shell Analysis of Guard Cell (GC) Deformations. *Transactions of the ASAE*, 19(6), 1107–1121) investigated the effect of various model constructs on the simulated pore. Such mechanisms tended to be internally consistent but could only explain trends and behaviours qualitatively, as inputs were idealised or estimated. (c) By constructing models based on real stomata, for example Yi *et al*. (2018) in Arabidopsis, quantitative models become tools used to measure aspects of the system. Keynia *et al*. (2023) compared simulated and experimental nanoindentations to concurrently estimate the turgor pressure and the anisotropic material properties of GCs. Yi *et al*. (2018) and Keynia *et al*. (2023) licenced under CC BY 4.0.

There are many reasons to build a model – to gain insight into and test understanding of a system, to generate predictions that guide experiments, or to infer properties of the system. These, in turn, dictate how data are employed in the model and likewise how its results are to be interpreted with respect to the features included. We have therefore attempted to classify existing stomatal models according to their purpose: descriptive, exploratory, and quantitative (Fig. 2).

The most elementary models are descriptive models, which may seem at first so basic as to be mere descriptions rather than models proper. However, we argue that these still perform the fundamental functions of models, namely in defining the scope of the problem, enumerating the features involved, and prescribing how they may interact to accomplish their function. The debt we owe to the descriptive models proposed by von Mohl (1856), Schwendener (1881), and Haberlandt (1914) cannot be overstated. Their writings contain the kernel of nearly every idea explored in stomatal mechanics to this day, including in this review. Schwendener, in particular, seems to have considered nearly every ‘intuitive’ way that stomata could open, including the most pervasive and enduring theory that ventral wall thickening causes stomatal opening. Schwendener also observed cross sections with uniform or dorsoventrally symmetrical thickenings, proposing both an equivalent of the modern ‘elongation’ mechanism and the typical Graminaceous (or dumbbell‐shaped) mechanism of polar end dilation. Later supplemented by Haberlandt (1914), he is also credited with a ‘constant width’ mechanism based on the circularisation of elliptical GCs. The illustrations shown in Fig. 2(a) from the widely used textbook ‘Physiology of Stomata’ by Meidner & Mansfield (1968) are largely based on these ideas.

But are these theories *correct*? Herein lies the main weakness of descriptive models – they are difficult both to ‘prove’ and to ‘use’ since they are formulated *post hoc* and do not involve simulation. This precludes making all but the most elementary predictions, in turn limiting their application to future experiments.

Simulating physical interactions between components of the stomate and the turgor pressure generated within the GC allows exploratory models to interrogate the feature space, finding the combinations needed to produce the expected results. The iconic physical model created by Aylor *et al*. (1973) explored the effect of various features such as a reinforced ventral wall and circumferentially oriented cellulose microfibrils (CMFs) by placing masking tape on inflated balloons. Their stated aim was to explore basic concepts ‘without being distracted by the anatomical details of a real stoma’. This was a double‐edged sword; these models are often impressively prescient, but it is precisely these ‘details’ that vary between stomata, so their omission precludes experimental verification. In creating the first finite element model (FEM) of stomata that simulates the interactions of a mesh of points representing the virtual GC's surface, Cooke *et al*. (1976) took a similar approach – in their own words: ‘in the absence of reliable experimental parameters for the model e.g., moduli of elasticity, Poisson's ratios and thickness, we are limited to an examination of trends’ (See Box 1, for a brief summary of these terms). These exploratory models thus build on prior descriptive models by testing the interactions between, and relative importance of, their features that could previously only be assumed to explain stomatal opening. These models are internally consistent, well‐closed loops in the simulation domain; however, due to their weaker connection to the empirical domain, they are more apt as hypotheses on how stomata *could* function, instead of assertions on how stomata *do* function.

Box 1 **Mechanical properties in plant biomechanics.**

This box provides basic explanations on relevant concepts in computational and mechanical details of modelling. Interested readers are referred to Bidhendi & Geitmann (2018) and references therein for further information.

**Mechanical properties:** mechanical properties describe a material's deformation behaviour in response to applied forces. The simplest model of a material is a Hookean solid, which is linearly elastic isotropic; that is, its properties do not vary with respect to the degree, rate, and direction of loading. Such a material's mechanics can be mathematically described entirely in terms of its Young's modulus and Poisson's ratio (will be defined later).

In reality, plant tissues violate this elementary model in nearly every conceivable way, the most important in the stomatal context being anisotropy (direction‐dependent stiffness). Advanced material models may accommodate some of these departures, but often at the cost of mathematical, conceptual, and computational complexity.

This difficulty extends to parameter measurement; since material properties are not directly observable, they must be inferred from empirical testing. Even basic mechanical experiments such as tensile tests and indentation rely on some type of model to relate measured forces and displacements to stress and strain within the material being tested. To a first order, these are mostly based on Hookean solids and, therefore, for plant tissues inherently approximations. Most researchers acknowledge that the values obtained are therefore, at best, estimates.

**Young's modulus:** this describes a material's stiffness or elasticity, independent of its shape. It is classically defined as the ratio of stress (force per unit area of material transverse to force) and strain (extension relative to original length in the direction of force).

**Poisson's ratio:** the Poisson's ratio relates to the fact that a strip of typical material becomes thinner and narrower when stretched lengthwise, or vice versa. Mathematically, this is expressed as the negative ratio of transverse and axial strains. An incompressible material has a Poisson's ratio of 0.5, such that its overall volume is preserved when it is stretched or compressed.

In the decades following Cooke's model, progress in stomatal mechanics was limited. However, the advent of cheaper, accessible, and more powerful computational methods, advances in technology to image stomata and to measure physical parameters of relevance to cell mechanics, along with a range of tools to manipulate stomatal systems via molecular genetics, has led to a resurgence of interest and advances in this area. A defining characteristic has been a movement away from demonstrating the opening of hypothetical stomates towards representing real, individual, stomates, or populations of stomata. These quantitative models are not simply more realistic – instead, this shift enables inference into properties of the system, most desirably those least accessible to direct measurement, such as cell wall mechanical properties or turgor pressure (Marom *et al*., 2017; Woolfenden *et al*., 2017; Yi *et al*., 2018; Chen *et al*., 2021; Keynia *et al*., 2023; Jaafar *et al*., 2024). However, such inverse models must close the inference loop completely, placing stricter demands on their conceptual ‘correctness’. The predictive power one stands to gain is matched by the risk of an incorrect or underpowered interpretation, which compromises the internal consistency of the model and calls its inferences into question. In short, the model is only as strong as its weakest link.

These models, though falling along historical lines, must be understood as building upon, rather than superseding, one another. The process of understanding the system is one of successive iteration between these model types rather than the creation of an ‘ideal’ model of any type.

**Fig. 3:**
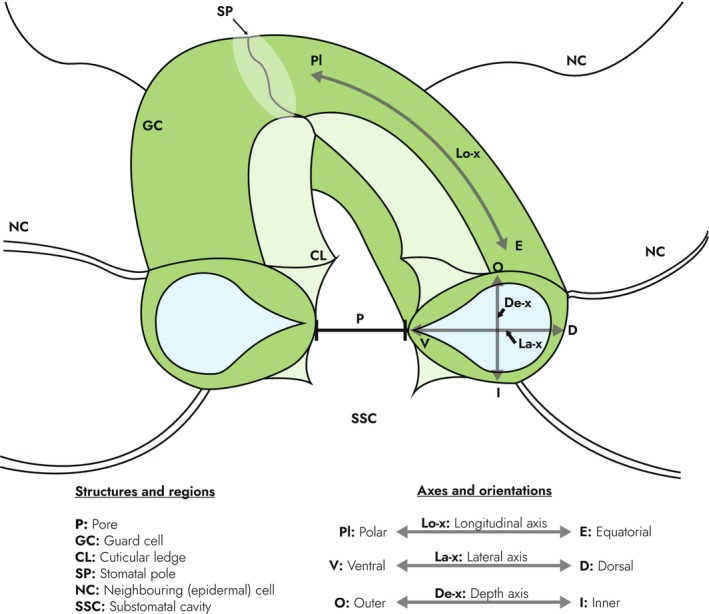
Schematic diagram of a typical eudicot (kidney‐shaped) stomatal complex. The left half is labelled with structures relevant to this review, while the right half delineates three anatomical axes used to navigate the stomate. Note that these are not always standardised within the literature.

Stomata are complex structures, and the study of their mechanics is spatially demanding. Fig. 3 establishes the anatomical structures and terminology of a typical eudicot ‘kidney‐shaped’ stomate to be used in this review.

## An appraisal of features in present models of stomatal mechanics

### Guard cell wall mechanical properties and cell elongation

All plant cells are surrounded by a cell wall that withstands the turgor pressure generated by osmotic differences with its environment, thus determining cell shape. The GC's unique shape and how it changes with inflation are therefore intrinsically linked with the cell wall's mechanical properties, which vary according to the interactions of its constituent polymers.

Primary cell walls (typical of GCs) comprise three main classes of polymers: cellulose, pectins, and hemicelluloses. CMFs are fibrous bundles of cellulose embedded in a matrix of pectins and hemicelluloses (Cosgrove, 2022). The high axial tensile modulus of CMFs relative to their softer embedding matrix makes the cell wall stiffer along the direction they are aligned, a property known as mechanical anisotropy (Baskin, 2005; Cosgrove, 2024). The arrangement of CMFs in GCs has been studied using a range of methods including polarised light microscopy (Ziegenspeck, 1955; Palevitz & Hepler, 1976; Shtein *et al*., 2017), fluorescent dyes (Rui & Anderson, 2016; Durney *et al*., 2023; Wilson *et al*., 2025), transmission electron microscopy (Palevitz & Hepler, 1976; Wille & Lucas, 1984), and field emission scanning electron microscopy (FESEM) (Fujita & Wasteneys, 2014).

Due to necessary compromises between sample preparation, specificity, and resolution, no single approach is sufficient to form a complete picture of CMF structure. For example, although FESEM's resolution visualises individual fibres in stunning detail, it does not conclusively indicate whether they are CMFs or some other cell wall polymer (Jaafar & Anderson, 2024). The ‘cartoon’ (i.e. conceptual) depiction of CMF arrangement as being ‘radially’ arranged about the central pore, and the corollary that they wrap around the circumference of GCs, is well‐established and universally accepted (Woolfenden *et al*., 2018; Jaafar & Anderson, 2024). However, it is rarely emphasised that it is an assembly of a range of empirical observations and may not fully capture dynamic reality. We believe this is consequential for its implementation in models.

Nearly all FEMs, with one notable exception (Cooke *et al*., 1976), converge on the conclusion that radial anisotropy (determined via CMF arrangement) is essential for the performance of the stomatal model (Aylor *et al*., 1973; Marom *et al*., 2017; Woolfenden *et al*., 2017). This radial anisotropy is explained to make GCs stiffer in cross section than along their length, such that they preferentially elongate when stretched by turgor pressure (Woolfenden *et al*., 2018; Yi *et al*., 2019; Anderson, 2023; Jaafar & Anderson, 2024). This tends to increase the length of the stomatal complex, and fixed complex length was found not to be essential in these models (Woolfenden *et al*., 2017). However, constant complex length (measured in the empirical domain) was the largest discrepancy between these models and observed stomatal behaviour, with Carter *et al*. (2017) imposing constant complex length as a boundary condition to both simulate reality and, at the same time, increase pore opening across the entire turgor range.

**Fig. 4:**
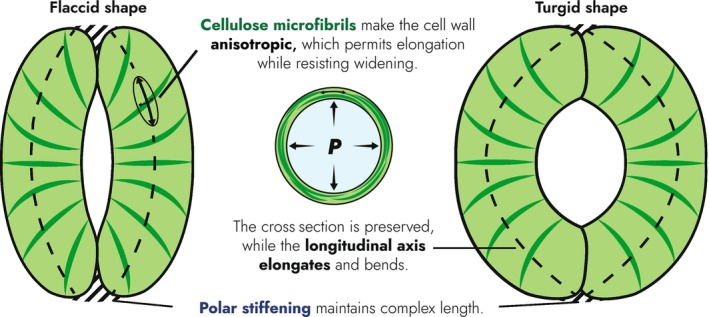
Elongation mechanism connecting guard cell (GC) elongation and pore opening. The central feature is cellulose‐derived anisotropy, represented by the strain ellipse showing direction‐dependent extension. This elongates the GC, bending it, while preventing its cross section from widening and inflating into the pore. This effect is accentuated if the complex length is prevented from increasing, for example by polar stiffening.

The resultant elongation mechanism (Fig. 4) clearly predicts that GC elongation drives stomatal opening. However, the actual empirical support for this is limited. Meckel *et al*. (2007) used 3D confocal imaging to demonstrate that while GCs do elongate during pore opening, they also widen significantly. As noted previously, both von Mohl (1856) and Schwendener (1881) observed that (at least in some stomata) changes in GC width occurred during changes in pore aperture, the inference being that concomitant shifts in cell depth were occurring; this is also illustrated in Fig. 2(a) and discussed in detail in the ‘Guard cell shape’ section. Although the technology of their time precluded the capture of 3D data to support this hypothesis, the pioneering data of Meckel *et al*. (2007) bring these ideas into the modern era.

The widening of GCs, while antithetical to the elongation mechanism, is not sufficient to discount it; both may be involved in the effective control of pore aperture. More compelling evidence is provided by Yi *et al*. (2018) who investigated the mechanical function of the three canonical cell wall polymers (cellulose, pectins, and xyloglucans) by studying stomatal movements in relevant *Arabidopsis* mutants. All mutants displayed altered but nevertheless functional stomatal behaviour. As expected, GCs in the wild‐type control stomata were found to elongate by *c*. 20%. Surprisingly, however, significant elongation was not observed in both the cellulose synthesis mutant *cesa3je5* (Desprez *et al*., 2007) and the xyloglucan‐deficient mutant *xxt1xxt2* (Cavalier *et al*., 2008). These findings provide clear evidence that GC elongation is not essential for stomatal function, implying that other elements of GC shape change are involved.

The data from this study also exemplify the difficulties inherent in connecting cell wall structure with stomatal function. While it is generally accepted that CMF alignment directs mechanical anisotropy, determinants of the extent of this anisotropy are much less clear (Baskin, 2005). Similarly, although pectin has been widely implicated in stomatal function and development (Jones *et al*., 2003, 2005; Rui *et al*., 2017, 2018; Chen *et al*., 2021; Carroll *et al*., 2022), debate continues over exactly how it impinges on wall mechanical properties (Bidhendi & Geitmann, 2018; Cosgrove, 2022). This limits our ability to relate changes in pectin chemistry to mechanical properties. The most common approach is to associate it with the cell wall's matrix stiffness, but how this happens at a mechanistic level remains conjecture. It is likely that pectin plays complex roles in determining cell wall structure through its interaction with, and patterning of, cellulose, but limitations in both the empirical and conceptual domain significantly constrain our understanding. Recent advances in our general understanding of plant cell wall structure/function (Zhang *et al*., 2014, 2016, 2017, 2021; Yu *et al*., 2024a,b) provide a foundation for advances with respect to GCs.

Finally, as an example of how empirical observations can foment new conceptual features, Rui & Anderson (2016) observed that the ‘image anisotropy’ of GCs stained with Pontamine Fast Scarlet 4B (a cellulose‐binding, orientation‐sensitive, fluorescent dye) decreases as stomata open. This was interpreted as a change in anisotropy of the CMFs, leading them to suggest that CMFs might bundle more tightly when the pore is closed, and unbundle when opened (Rui *et al*., 2018; Anderson, 2023; Jaafar & Anderson, 2024). This is an intriguing idea that warrants further investigation.

To summarise, our appraisal of the evidence is that while GC elongation certainly accompanies stomatal movement, with CMF alignment key to this process, it is unlikely to be the only significant driving force behind pore aperture change. The nature and relative importance of alternative aspects of GC expansion/shape change await full elucidation. This uncertainty reflects a broader lack of clarity on the control of GC wall anisotropy and its role in stomatal mechanics.

### Guard cell shape

A deeply embedded idea in stomatal literature (both in textbooks and in online resources) is that bending of GCs is the result of a difference in stiffness, and thus in extension, of a thick ventral wall relative to its opposing dorsal wall. The earliest mention of this idea we could find in English is from Haberlandt (1914), who attributes it to his mentor, Schwendener, likely in 1881.

**Fig. 5:**
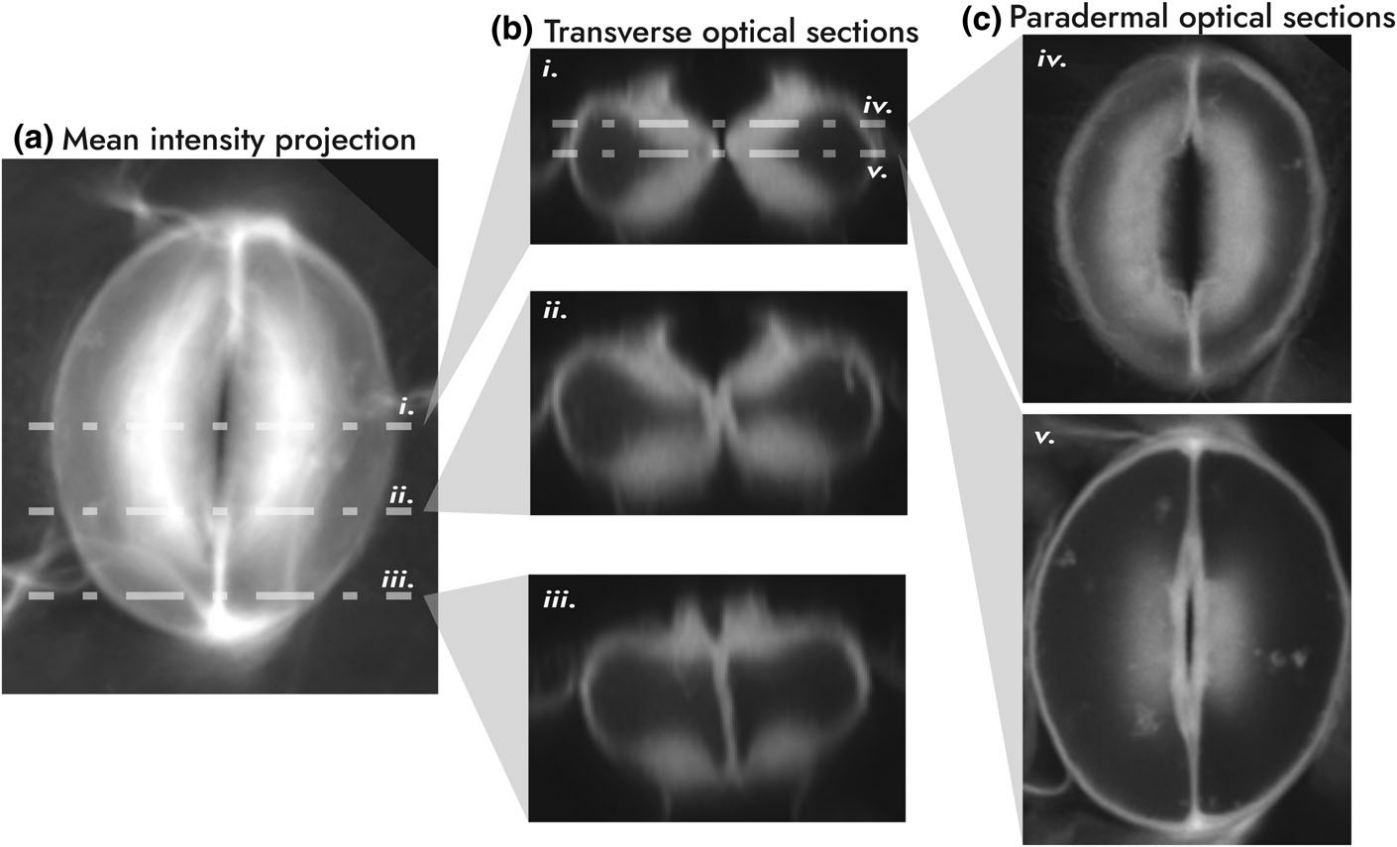
Visualisation of guard cell shape. To demonstrate the perils of perspective in studying stomatal shape, an *Arabidopsis* stomate was imaged in 3D with confocal microscopy using propidium iodide, a cell wall stain, and projected onto different planes. (a) A mean intensity projection approximates how it would appear from above using top‐down light microscopy. The positions of typical transverse sections are shown (i–iii). Note the appearance of the thickened ventral wall. (b) (i) Sectioning the stomate across the equator produces the stereotypical cross section. Note the extent of upper and lower wall thickening, which gives the enclosed protoplast a triangular shape. This pattern is greatly altered towards the poles (sections ii–iii). (c) Paradermal optical slices at different positions (iv, v) further emphasise the appearance of a thickened ventral wall. Image data courtesy of Dr Matt Wilson, University of Sheffield, collected as per Wilson *et al*. (2025).

Over time, an important subtlety to Schwendener's original explanation seems to have been lost. He was also among the first to illustrate the complex cross section of typical kidney‐shaped GCs, depicted in Fig. 5(b,i). The ventral wall is on average indeed much thicker than the opposing dorsal wall, but its thickenings are concentrated in the upper and lower quadrants, whereas the innermost ventral region (i.e. at the midsection of the pore) may be as thin as the dorsal wall, creating a triangular shape to the enclosed GC protoplast. Schwendener (1881; via Haberlandt, 1914) suggested that this shape supplemented stomatal opening by analogy with the ‘hinges of a portfolio’.

Interestingly, when viewed from above (Fig. 5c,iv) or in paradermal sections (Fig. 5c,v) the ventral wall gives the impression of being uniformly thickened. This simplified geometry was incorporated into several computational and physical models, which simulated a difference in ventral/dorsal wall thickness or stiffness. Such studies unanimously showed it had minimal impact on model stomatal performance, especially compared with the apparent effectiveness of cellulose‐derived anisotropy. As a result of this simplification in modelled geometry, Schwendener's ‘portfolio’ hypothesis remained untested.

Investigators have more recently modelled stomata from actual cross sections with more realistic thickenings (Pautov *et al*., 2017, 2019, 2024; Yi *et al*., 2018; Chen *et al*., 2021; Keynia *et al*., 2023; Jaafar *et al*., 2024). While these models did not explicitly explore their effect on stomatal performance, changes in the cross‐sectional shape were observed to accompany changes in cell wall mechanical properties and cell geometry, both across GC age (Jaafar & Anderson, 2024) and in mutants with altered cell wall polymer composition (Yi *et al*., 2018; Keynia *et al*., 2023). These data support the potential relevance of GC cross‐sectional shape to stomatal function, but the mechanistic link still awaits confirmation.

A further potential significance for the noncircular shape of GCs in stomatal pore movement was proposed via Schwendener's ‘constant width’ mechanism (Fig. 2a). This idea was further developed by Sharpe & Wu (1978), who suggested that a biphasic opening dynamic, first reported by Stålfelt (1927), is achieved by the circularisation of an initially elliptical or triangular cross section in the ‘*Spannungsphase*’ (tension‐phase) and completed in the ‘*Motorischephase*’ (motor‐phase). It is interesting to note that this same trend later led Woolfenden *et al*. (2017) to propose that the GC wall strain‐stiffens. Cooke *et al*. (1976) similarly found that a ‘doubly elliptical’ shape, in which both cross section and longitudinal axis are semicircular, was sufficient to achieve stomatal opening independent of other system parameters (e.g. CMF anisotropy). Furthermore, a circularisation of the GC lumen is visible to some extent in the four phylogenetically diverse examples examined in Franks & Farquhar (2007) and possibly also in *Vicia faba* (Meckel *et al*., 2007), suggesting it may not be confined only to *Mnium* and *Helleborus* as reported in the century prior (Schwendener, 1881; Haberlandt, 1914). These observations, somewhat forgotten in more modern literature, raise the tantalizing possibility of a supplementary or parallel mechanism to those elucidated so far, supporting our suggestion that GC elongation is not the entire story behind stomatal pore opening.

To conclude this section, stomatal mechanics has, through simulation, established that a simple mechanism based on a dorsoventral thickness difference is insufficient to open the pore. However, in revisiting the empirical and conceptual basis for this mechanism, it is hopefully clear how this misrepresents the cross‐sectional shape of GCs, whose actual, more complex, variable wall thickness remains wide open for further investigation. Efforts in all three domains – beginning with improved quantification of GC shape changes during stomatal pore opening, followed by thoughtful conceptual interpretation and simulation – are needed to provide a deeper understanding of this long‐studied but still somewhat contentious area of stomatal mechanics.

Differential wall thickening and cross‐sectional shape are two elements of GC shape that have received the most attention in models of stomatal mechanics. There are clearly other elements that, to date, have received very limited study, for example cuticular ledges and peristomatal rings (Pautov *et al*., 2017, 2019). At a larger scale, parameters such as the ratio of pore length to GC junction length (proposed as developmental landmarks in stomatal shape by Jaafar *et al*. (2024)) may also influence stomatal function via their influence on overall complex geometry (Wu *et al*., 1985). This aspect of GC shape (involving interactions with surrounding cells) overlaps with the concept of mechanical advantage, considered in the ‘Mechanical advantage and the role of neighbouring cells’ section.

### Mechanical advantage and the role of neighbouring cells

GCs are surrounded by other epidermal cells, which clearly have a mechanical influence on stomata. In this review, we define these by the general term ‘neighbouring cells’ (NCs), also known as ‘peristomatal cells’ (Fig. 3). In the z‐direction, GCs lie suspended over the substomatal cavity, so in physical terms, the mechanical interactions between GCs and NCs are primarily within the plane of the epidermis. The antagonism between GCs and NCs have been pondered since the field's inception (von Mohl, 1856) and has predominantly come to be conceptualised in terms of ‘mechanical advantage’.

Of particular interest in the early stomatal literature was the anomalous Iwanoff effect (the ‘wrong‐way response’), in which conditions provoking stomatal closure initially cause a transient opening response. Such conditions include excision (Iwanoff, 1928), lowered hydrostatic pressure in the water supply to a leaf (Raschke, 1970), or a flow of dry air over a leaf (Mott & Franks, 2001). The theory of mechanical advantage posits that pore size is somehow more reactive to changes in epidermal turgor than GC turgor. Therefore, when an external environmental stimulus provokes a change in turgor in the same direction in both cells, the initial ‘hydropassive’ response is first dominated by the NC, while the later ‘hydroactive’ regulation of turgor by ion fluxes subsequently provides the expected GC‐dominated response (Buckley, 2005; Roelfsema & Hedrich, 2005).

However, unfortunate tautology aside, the actual mechanics of ‘mechanical advantage’ have yet to be satisfactorily resolved. The first and most enduring explanation revolves around the relative size of GCs and NCs. Since total force is the product of turgor pressure and area, equivalent total forces may be obtained from a smaller epidermal turgor acting over a larger area (DeMichele & Sharpe, 1973; Wu *et al*., 1985). A geometric interpretation, offered by Wu *et al*. (1985), specifies the relevant dimensions to be the length of the stomatal apparatus, pore length, and the relative epidermal cell contact depth. Another popular explanation focusses on ‘mechanical interaction’, roughly, the extent of the shared wall in cross section (Franks & Farquhar, 2007). An intricate modelling effort by Pautov *et al*. (2024) further suggests that the mutual alignment of GCs and NCs exerts a strong influence on the degree of mechanical advantage.

These explanations, though appealingly intuitive, remain largely at the descriptive and exploratory level. An interesting exception is illustrative of this problem – evidence from physiological studies has suggested a reversal of mechanical advantage in ferns (Brodribb & McAdam, 2017). Their GCs are indeed larger than their NCs (Pichaco *et al*., 2024), corroborating the cell‐size theory, but the litany of other physical factors described previously has yet to be considered. How does the interplay between all these factors produce mechanical advantage? The application of simulations should provide routes to bridge this gap, but most modern models incorporate mechanical advantage in a somewhat rudimentary fashion.

For example, Cooke *et al*. (1976) and Woolfenden *et al*. (2017) apply a back pressure on the external surface of stomata, with the former even plotting it as a function of the ‘antagonism ratio’ (an analogue to mechanical advantage). However, these representations do not encompass the notion that epidermal cell geometry influences mechanical advantage. This is exemplified in the latter model in which the pore only opens once GC turgor exceeds the imposed back pressure; this implies that epidermal–GC turgor relations are reducible to a net turgor evaluation, which is not correct (DeMichele & Sharpe, 1973; Glinka, 2006).

This is not to say that modern FE models disregard NCs entirely. Many models represent a specific role for NCs relating to the poles of the stomatal complex (Fig. 3), which have been observed to be stationary during stomatal movement (von Mohl, 1856; Schwendener, 1881; Meidner & Mansfield, 1968). A study by Carter *et al*. (2017) spurred a renewed emphasis on this restriction. The authors observed an increase in Atomic Force Microscopy (AFM) indentation modulus at the poles of the stomatal complex, which they termed polar stiffening. Relating this to earlier observations of methylesterified pectin accumulation in a similar region (Amsbury *et al*., 2016), they reasoned that these stiff poles correspond to a ‘pinning’ to their polar NCs, restricting stomatal complex lengthening. Similarly, Yi *et al*. (2018) imposed geometric boundary conditions based on observed changes in stomatal complex dimensions. Both found that these restrictions increased the extent of pore opening compared with equivalent unrestricted models. On this basis, it has become commonplace to impose boundary conditions confining the poles and/or restricting displacement of the dorsal GC edges (Marom *et al*., 2017; Yi *et al*., 2019; Chen *et al*., 2021; Keynia *et al*., 2023; Jaafar *et al*., 2024).

Recent notable empirical studies provide evidence that challenges the straightforward association between polar stiffening and complex length restriction of initial models. First, Zheng *et al*. (2023) found that in poplar mutants with decreased polar stiffening, complex length still remained constant during stomatal opening. Pores instead opened wider, with GCs occasionally even completely separating at the tips. Second, Davaasuren *et al*. (2022) measured stomatal geometry changes caused by ablating permutations of junctional (i.e. polar) and flanking epidermal cells in *Arabidopsis*. Ablation of all surrounding cells, not just polar cells, was needed to elicit a significant change in complex length, and unexpectedly caused them to *shorten* rather than *lengthen*. Instead, ablating only polar cells led to slight but significant widening of the complex, corroborating the findings described previously. These suggest that polar stiffening is not wholly responsible for (and may indeed be unrelated to) the maintenance of stomatal complex length, that both polar and lateral epidermal cells may be involved in maintaining complex length, and that, under certain conditions, there is an inherent tendency for stomata to shorten, contrary to the standard elongation mechanism.

In summary, there remains a mismatch between our conceptualisation of mechanical advantage and the empirical behaviours it relates to (e.g. the Iwanoff effect). Even the ‘simple’ cases of NCs providing mechanical support and restriction remain challenging to model and experimentally verify. There thus remains ample room for progress.

## Conclusion

**Fig. 6:**
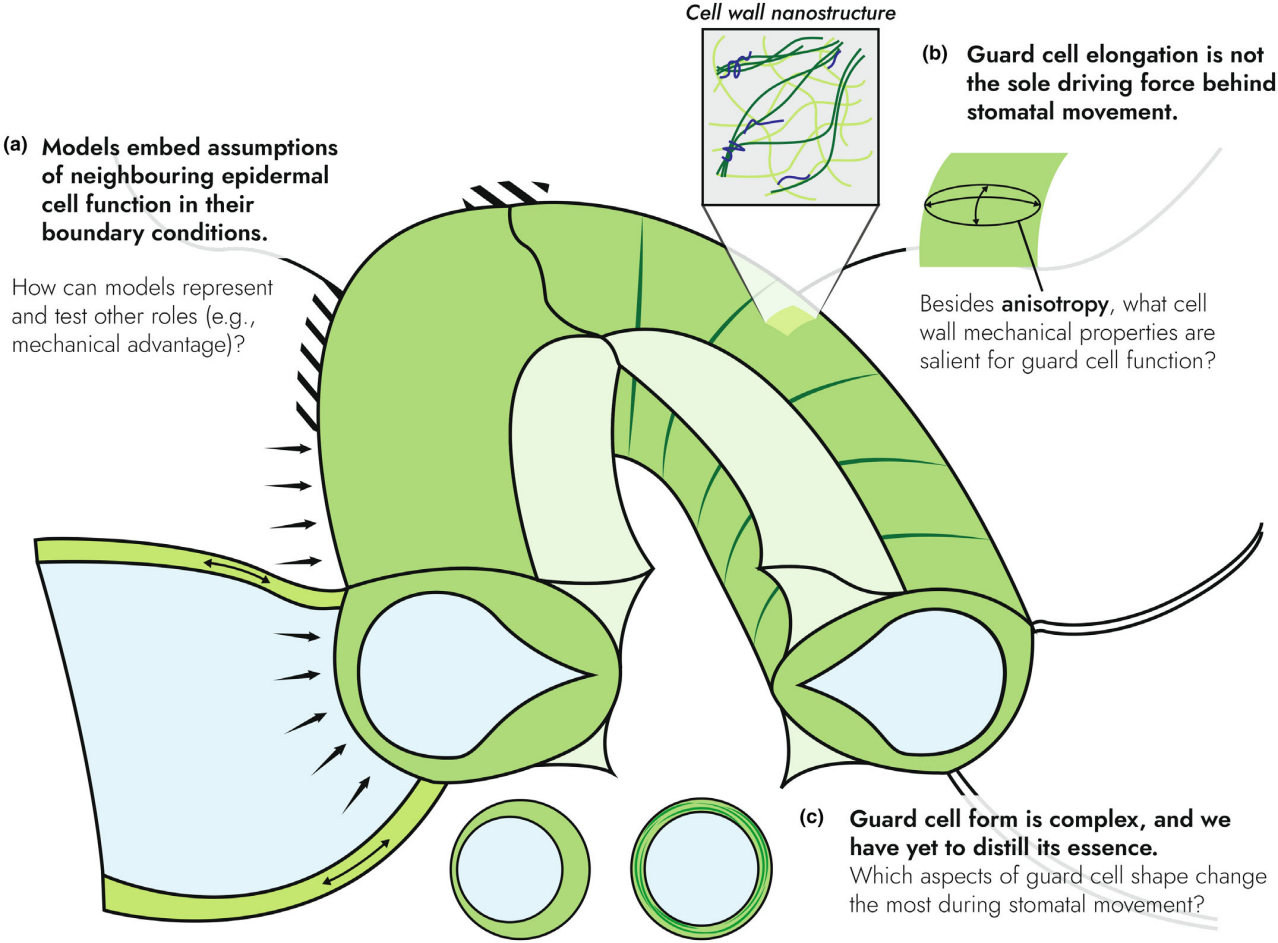
Most pressing issues and questions in stomatal mechanics. (a) Whereas mechanical advantage is viewed as essential to guard cell (GC)‐neighbouring cell relations in the wider stomatal mechanics literature, modern finite element models have simplified the role of epidermal cells to polar and lateral fixation (stripes), or direct back pressure (arrows), which are fundamentally at odds with this idea; this could involve, for example, the epidermal cell wall's own axial tension (double‐headed arrows). (b) The certainty with which anisotropy‐driven elongation is thought to explain stomatal movement is at odds with the nascency of our understanding of the cell wall's complex nanostructure from which continuum‐scale mechanical properties emerge (represented by the latest ‘biomechanical hotspot’ model, after Cosgrove (2024)). The resulting complex mechanical behaviours are likely to be relevant for stomatal mechanics. (c) The study of GC shape was once confined to two‐dimensions, leading to simplifications in shape and structure such as the ‘thickened ventral wall’ or ‘hoop‐stiffening’ of the cross section. These idealisations are no longer necessary nor adequate to explain stomatal behaviour. The questions of which shape changes occur during stomatal movements is fundamental to stomatal mechanics and should be reassessed with the greater spatiotemporal resolution afforded by modern methods.

Despite the clear advances that have been made over the last decade, weaknesses remain in extant mechanical models of stomata, limiting our insight into how the systems work. Fig. 6 highlights the essential limitations and open questions prompted by this critical analysis. We have organised the review around individual structures and features, but it is hopefully clear that these are interconnected in complex ways. A challenge for future work is to meaningfully combine these features, especially when they bridge scales (nano‐ to meso‐ to cell). Although the extant models have been very useful in providing mechanistic insight, they all incorporate various assumptions needed for the models to ‘work’. It seems possible that these models are missing some key elements, as if we were assembling a puzzle without all the necessary pieces. Finding these missing pieces will require better empirical data on actual cell geometry and wall properties, preferably incorporating time‐dependent behaviour. The latest quantitative models in the field hold great potential and have already enabled inference of complex quantities such as time‐dependent or anisotropic mechanical properties (Chen *et al*., 2021; Keynia *et al*., 2023). However, the extent to which such inferred quantities can safely be used as priors in future models remains an open question, for which experimental validation of biomechanical parameters remains essential.

Finally, advanced models already exist that capture the complex regulation of ion fluxes in GCs that drive the turgor pressure changes underpinning stomatal mechanics (Chen *et al*., 2012; Nguyen *et al*., 2023). Combining such flux‐based models with biomechanical models would provide an integrated understanding of how stomata work and could be an exemplar for modelling biology across scales.

## Competing interests

None declared.

## Author contributions

NYHT wrote the first draft with JVA, with JEG and AJF providing input. All authors contributed to the final version.

## Disclaimer

The New Phytologist Foundation remains neutral with regard to jurisdictional claims in maps and in any institutional affiliations.
